# Rapid development of an online tracker to communicate the human impact of abruptly halting PEPFAR support

**DOI:** 10.1002/jia2.26433

**Published:** 2025-02-26

**Authors:** Brooke E. Nichols, Elvin H. Geng, Eric Moakley, Andrew N. Phillips, Jeffrey W. Imai‐Eaton, John Stover, Edinah Mudimu, Anna Grimsrud

**Affiliations:** ^1^ Department of Global Health Boston University School of Public Health Boston Massachusetts USA; ^2^ Department of Global Health, Amsterdam Institute for Global Health and Development Amsterdam UMC, University of Amsterdam Amsterdam the Netherlands; ^3^ Infectious Diseases Division, Institute for Public Health Washington University in St. Louis St. Louis Missouri USA; ^4^ End with Something Amsterdam the Netherlands; ^5^ Institute for Global Health University College London London UK; ^6^ Center for Communicable Disease Dynamics, Department of Epidemiology Harvard T.H. Chan School of Public Health Boston Massachusetts USA; ^7^ MRC Centre for Global Infectious Disease Analysis, School of Public Health Imperial College London London UK; ^8^ Avenir Health Glastonbury Connecticut USA; ^9^ Department of Decision Sciences University of South Africa Pretoria South Africa; ^10^ HIV Programmes and Advocacy IAS‐the International AIDS Society Cape Town South Africa

1

On 24 January 2025, the global HIV community was confronted with the abrupt announcement that the U.S. President's Emergency Plan for AIDS Relief (PEPFAR), a programme with longstanding bipartisan support, would be paused for 90 days following an executive order to halt all foreign aid [1, 2]. For the over 20 million individuals worldwide receiving HIV treatment through PEPFAR‐supported programmes, the potential consequences were severe and immediate. As the public health community sought mechanisms to respond, an accurate, quantitative and science‐based understanding of the potential magnitude of this pause was urgently needed. Systematic quantification of the potential impacts would allow the public health community to plan harm reduction strategies, advocate for policy responses and effectively communicate the severity of the situation to stakeholders, including government officials, civil society leaders, media outlets and advocacy groups.

To provide a rapid assessment, we undertook a near real‐time modelling effort. We leveraged existing modelling work, publicly available data and expert consensus to generate projections. Complex mathematical modelling typically requires weeks to generate robust projections, limiting its utility for real‐time decision‐making. Our focus was on answering the most pressing question—the potential health consequences of this funding pause—using the most parsimonious model possible.

The fundamental parameters necessary for this assessment were drawn from public sources and included:

- The number of individuals receiving HIV treatment through PEPFAR support, using publicly available data from PEPFAR‐supported agencies, including the U.S. Centers for Disease Control and Prevention and USAID [3, 4].
- The estimated health and HIV transmission consequences of a 90‐day treatment disruption, applying established disability‐adjusted life years values associated with treatment interruptions [5].
- The projected number of infant deaths resulting from a 90‐day interruption in antiretroviral therapy (ART) for pregnant women and their newborns, based on published data on maternal and neonatal HIV outcomes and PEPFAR programme data from amfAR [3, 6, 7, 8, 9].

To facilitate accessibility and dissemination, we collaborated with a product development expert to translate these calculations into an interactive website, now available at https://pepfar.impactcounter.com/. The site launched on 28 January 2025, 4 days after the funding freeze was announced [10]. The platform provides an intuitive tool for users to quickly understand the potential consequences of the PEPFAR funding freeze.

As the tool gained visibility and traction, we continued to refine the modelled estimates through additional expert review. Members of the HIV modelling community, including those affiliated with the HIV Modelling Consortium, independently assessed the calculations, refined assumptions and incorporated the latest available data. This collaborative process ensured that the estimates remained as accurate and reliable as possible while maintaining clarity in communication.

The current estimates on adult deaths presented on the website are informed by detailed modelling projections from five well‐established HIV mathematical models [11]. We used estimates from Jewell et al. on the number of excess deaths expected to occur over a 1‐year time horizon associated with complete ART service disruptions over a 90‐day period—as a reduction in ART is what specifically drives short‐term mortality. This number was multiplied by the percent of HIV programmes funded by PEPFAR (47%) [12]. We assumed a linear distribution of deaths over the year, resulting in one death every 3.3 minutes.

There may be a 1‐month lag between the funding freeze and when deaths begin, given the time required for viral rebound. The tracker, however, provides an understanding of the net current impact of the funding freeze. Additionally, these projections are short‐term, but the total long‐term impact is likely significantly greater due to onward HIV transmission resulting from service interruptions—and given the time required to re‐establish services and re‐engage those who have disengaged from the health system. Once service availability is clearer, the increment of deaths will be adjusted accordingly. Modelling the number of new infections from specific prevention and treatment disruptions caused by the funding freeze will become increasingly important.

As of 19 February 2025, the tool had undergone 127 updates, incorporating new insights, improved assumptions and user feedback. This iterative refinement process was made possible by the concerted and collaborative efforts of HIV modellers, clinicians, public health professionals and web developers. It has also been updated in response to direct requests from U.S. congressional staffers to add additional information and impact numbers. Despite the lack of mainstream media coverage, the website has been viewed more than 18,000 times across 153 countries.

The development of this rapid response tool has yielded six key lessons:

1. **Timeliness is critical**: Decision‐making in emergency situations often proceeds without comprehensive evidence. Rapidly generating and disseminating essential data can inform policy discussions and advocacy efforts.
2. **Clear communication enhances impact**: Simplifying complex modelling outputs into accessible and interpretable formats enhances their utility for policymakers and advocates.
3. **Collaboration strengthens methodology**: Engagement with the HIV modelling community and interdisciplinary experts improved the credibility and accuracy of the estimates.
4. **Transparency fosters trust**: Documenting methodological updates and acknowledging the limitations of our calculations increased confidence in the results and encouraged constructive feedback.
5. **An iterative process is effective**: Continuous refinement of estimates, rather than delaying dissemination until a “perfect” model is achieved, ensured timely and credible information was available as new data emerged.
6. **Digital tools amplify reach**: A web‐based platform enabled broad dissemination and engagement, enhancing the visibility and impact of the findings.

At present, the tracker provides estimates based on well‐characterized impacts of ART interruptions. As more data become available on the “known unknowns,” further refinements will be incorporated. Key areas of (un)certainty include:

*The known knowns*:

- The abrupt cessation of funding has had, and will continue to have, significant repercussions for HIV programmes in PEPFAR‐supported countries.
- Treatment interruptions will contribute to HIV‐related morbidity, mortality and new HIV infections, resulting in long‐term human and economic costs and consequences.

*The known unknowns*:

- The extent of healthcare workforce disruptions in PEPFAR‐supported countries and their impact on HIV services.
- The implications of waivers issued to continue select services and ongoing legal challenges to the funding freeze.
- The timeline and extent of programme resumption if funding is effectively reinstated.
- Empiric estimates of ART interruptions due to the funding freeze.
- The potential erosion of trust in healthcare services, affecting future engagement in HIV care.
- How, and how effectively, national HIV programmes will respond to mitigate the short‐ and long‐term impacts of the stop‐work order.

*Future unknowns*:

- The broader impact on other health services due to the reallocation of healthcare workers to mitigate HIV impact.
- Long‐term economic consequences of the funding freeze, of the freeze on the willingness of other donors to continue supporting foreign aid, and the economic impact of an increase in HIV cases and HIV‐related mortality.
- The extent to which the halt in PEPFAR funding may undermine progress towards the goal of ending AIDS as a public health threat.

As the situation evolves, and there are updates on the delivery of HIV services, the trackers will continue to be refined with the support of the HIV Modelling Consortium. The online tool serves as a dynamic resource to inform advocacy, policy decisions and global HIV response efforts. Although given rapid policy shifts, the future is not knowable, modelling provides a science‐based projection of the consequences of current policy on systems and social conditions. This can give the public health community a shared sense of the magnitude, timing and nature of the crises, enabling collective action.

## COMPETING INTERESTS

The authors declare no competing interests.

## AUTHORS’ CONTRIBUTIONS

1

BEN conceptualized and developed the first draft of this Field Note. EHG, ErM, ANP, JWI‐E, JS, EdM and AG provided guidance, input and subsequent revision. All authors have reviewed and approved the final manuscript.

## Data Availability

Data sharing is not applicable to this article as no datasets were generated or analysed during the current study.

## References

[1] Reevaluating and realigning United States foreign aid—The White House. [cited 11 Feb 2025]. Available: https://www.whitehouse.gov/presidential‐actions/2025/01/reevaluating‐and‐realigning‐united‐states‐foreign‐aid/

[2] Exclusive: State Department issues stop‐work order on US aid | Devex. [cited 11 Feb 2025]. Available: https://www.devex.com/news/exclusive‐state‐department‐issues‐stop‐work‐order‐on‐us‐aid‐109160

[3] AIDSinfo | UNAIDS. [cited 11 Feb 2025]. Available: https://aidsinfo.unaids.org/

[4] Results and impact—PEPFAR—United States Department of State. [cited 11 Feb 2025]. Available: https://www.state.gov/results‐and‐impact‐pepfar

[5] Bershteyn A , Jamieson L , Kim HY , Platais I , Milali MP , Mudimu E , et al. Transmission reduction, health benefits, and upper‐bound costs of interventions to improve retention on antiretroviral therapy: a combined analysis of three mathematical models. Lancet Glob Health. 2022;10:e1298–e1306. 10.1016/S2214-109X(22)00310-2 35961353 PMC9380252

[6] Violari A , Cotton MF , Gibb DM , Babiker AG , Steyn J , Madhi SA , et al. Early antiretroviral therapy and mortality among HIV‐infected infants. N Engl J Med. 2008;359(21):2233–2244. 10.1056/NEJMoa0800971 19020325 PMC2950021

[7] PEPFAR Monitoring, Evaluation, and Reporting Database. [cited 11 Feb 2025]. Available: https://mer.amfar.org/

[8] Flynn PM , Taha TE , Cababasay M , Fowler MG , Mofenson LM , Owor M , et al. Prevention of HIV‐1 transmission through breastfeeding: efficacy and safety of maternal antiretroviral therapy versus infant nevirapine prophylaxis for duration of breastfeeding in HIV‐1‐infected women with high CD4 cell count (IMPAACT PROMISE): a randomized, open label, clinical trial. J Acquir Immune Defic Syndr. 2018;77:383. 10.1097/QAI.0000000000001612 29239901 PMC5825265

[9] Dabis F , Fransen L , Halsey N , Lepage P , Msellati P , Newell ML , et al. Rates of mother‐to‐child transmission of HIV‐1 in Africa, America, and Europe: results from 13 perinatal studies. The Working Group on Mother‐To‐Child Transmission of HIV. J Acquir Immune Defic Syndr Hum Retrovirol. 1995;8:506–510. 10.1097/00042560-199504120-00011 7697448

[10] How GenAI tools enable rapid impact—by Eric Moakley. [cited 11 Feb 2025]. Available: https://substack.com/home/post/p‐156023174

[11] Jewell BL , Mudimu E , Stover J , ten Brink D , Phillips AN , Smith JA , et al. Potential effects of disruption to HIV programmes in sub‐Saharan Africa caused by COVID‐19: results from multiple mathematical models. Lancet HIV. 2020;7:e629–e640. 10.1016/S2352-3018(20)30211-3 32771089 PMC7482434

[12] UNAIDS HIV Financial Dashboard . [cited 19 Feb 2025]. Available: https://hivfinancial.unaids.org/hivfinancialdashboards.html

